# *Angiostrongylus cantonensis* Lungworms in Definitive and Intermediate Hosts, Madagascar, 2024

**DOI:** 10.3201/eid3110.241741

**Published:** 2025-10

**Authors:** Lanto A. Maminirina, Zaïna I. Bodoarison, Minoarisoa Rajerison, Séverine Ferdinand, Beza Ramasindrazana

**Affiliations:** Université de Fianarantsoa, Fianarantsoa, Madagascar (L.A. Maminirina); Unité Peste, Institut Pasteur de Madagascar, Antananarivo, Madagascar (L.A. Maminirina, Z.I. Bodoarison, M. Rajerison, B. Ramasindrazana); Université d’Antananarivo, Antananarivo (L.A. Maminirina, Z.I. Bodoarison, M. Rajerison, B. Ramasindrazana); Unité Environnement–Santé, Equipe Ecologie Microbienne, Institut Pasteur de la Guadeloupe, Guadeloupe, France (S. Ferdinand)

**Keywords:** *Angiostrongylus cantonensis*, rat lungworm, parasites, rats, snails, Madagascar

## Abstract

We assessed the prevalence of the rat lungworm, *Angiostrongylus cantonensis*, in rats and snails in Toamasina, Madagascar, using molecular techniques. Although no human cases of neuroangiostrongyliasis have been reported in Madagascar, the pathogen’s presence in definitive hosts (2.5%, 2/78) and intermediate hosts (26.9%, 35/130) reveals active circulation and potential zoonotic risk.

The rat lungworm, *Angiostrongylus cantonensis* (Strongylida: Angiostrongylidae), first identified and described in China in 1935 (*1*), is responsible for neuroangiostrongyliasis, which can cause neurologic damage, such as eosinophilic meningitis or encephalitis, in humans. The life cycle of this parasitic nematode involves rats as definitive host and snails and slugs as intermediate hosts; humans act as incidental hosts only in this system (*2*).

Neuroangiostrongyliasis is a foodborne disease, contracted by humans through accidental ingestion of the *A. cantonensis* infective larvae; several thousand human cases have been documented worldwide (*3*). The infective larva can be found not only in intermediate hosts but also in paratenic hosts (e.g., crustaceans, frogs), on contaminated vegetables, and in water (*2*,*4*).

In Madagascar, *A. cantonensis* worms were first sampled in 1964 (*5*) in rats from Ambavaniasy, Alaotra Mangoro Region, and later confirmed in *Rattus* spp. rats (*6*). The parasite was reported in rats captured in Antananarivo in 1982 (*7*); prevalence was 12% during the rainy season and 5% during the dry season. Human cases of neuroangiostrongyliasis on the neighboring islands of Mayotte and La Reunion have been reported (*2*). In addition, human breeding and consumption of intermediate hosts of this parasite, specifically snails of the genus *Achatina*, are still taking place on the island; however, data on the potential role of this snail as a carrier of *A. cantonensis* worms are currently unavailable. In this context, the aim of this study was to update the occurrence of *A. cantonensis* worms in rats and investigate their presence in snails.

We trapped rats and collected snails in March 2024 in 3 communes in Toamasina district: Fanandrana, Antetezambaro, and Ankirihiry (Appendix). Further, we collected snails of the genus *Achatina* (Figure 1, panel A) and sampled and preserved a portion of the foot of each snail for molecular screening for the presence of *A. cantonensis* worms*.* After DNA extraction, we performed conventional PCR targeting the cytochrome c gene to confirm the identity of adult lungworms (Appendix). Further, we performed molecular screening of *A. cantonensis* worms using TaqMan quantitative PCR (Thermo Fisher Scientific, https://www.thermofisher.com) targeting the internal transcribed spacer gene (Appendix). We used the χ^2^ test to compare the statistical differences in intermediate host infection by localities.

**Figure 1:**
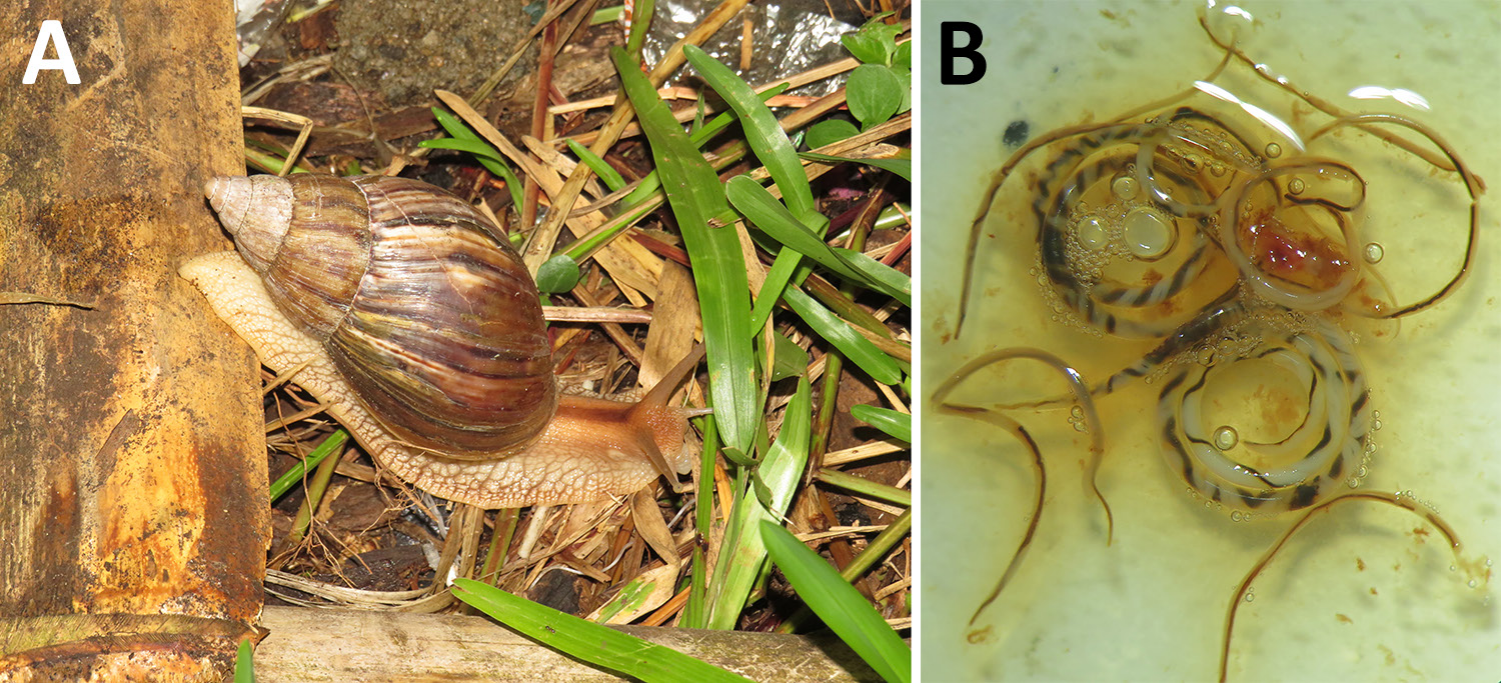
Specimens obtained in study of *Angiostrongylus cantonensis* lungworms in definitive and intermediate hosts, Madagascar, 2024. A) An *A. cantonensis* worm intermediate host giant African land snail (*Achatina* sp.) in Madagascar; B) adult male and female *A. cantonensis* worms extracted from the lung of an infected *Rattus norvegicus* rat.

We captured a total of 78 individual rats of 2 species, 76 *Rattus rattus* rats and 2 *R. norvegicus* rats; 61.5% (48/78) were male and 38.4% (30/78) were female, and the age distribution was 55.1% (43/78) juvenile and 44.8% (35/78) adult (Table 2; Appendix). Two *R. norvegicus* rats captured in Ankirihiry harbored 12 adult lungworms; 8 were isolated from a pregnant female rat and 4 from a juvenile male rat (Figure 1, panel B). We amplified and sequenced 2 adult lungworms from each of the 2 rats (GenBank accession nos. PV185895, PV185896). Those sequences showed 100% similarity with a partial genome sequence of an isolate from Spain (accession no. PP748576) and clustered with nucleotide sequences from Spain, Australia, the United States, Japan, and Brazil (Figure 2). The overall prevalence of infection in rats was 2.5% (2/78).

**Figure 2:**
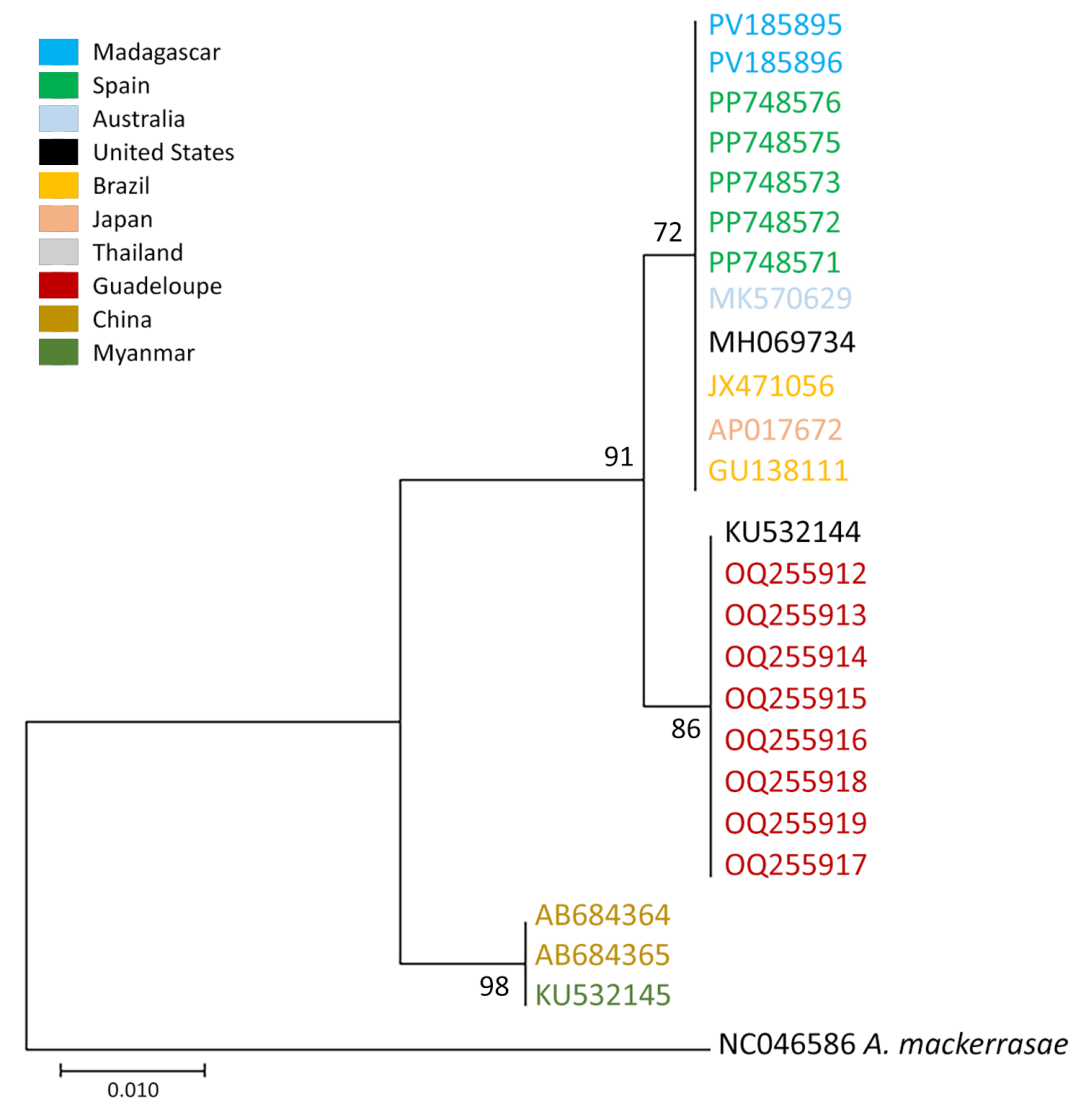
Phylogeny of adult *Angiostrongylus cantonensis* lungworm based on cytochrome c sequences in definitive and intermediate hosts, Madagascar, 2024. Labels are GenBank accession numbers, colored by location. The phylogenetic tree was constructed using the Tamura-Nei 1993 plus gamma distribution model with 10,000 iterations in MEGA X (https://www.megasoftware.net). All bootstrap values are shown on corresponding nodes. *Angiostrongylus mackerrasae* was used as the outgroup. Scale bar indicates number of substitutions per site.

For the intermediate host, we collected 130 snails of the *Achatina* genus. A total of 35 individual snails tested positive by quantitative PCR, reaching an overall prevalence of 26.9% (35/130); rates of prevalence were 51.5% (17/33) in Ankirihiry, 29.6% (16/54) in Antetezambaro, and 4.6% (2/43) in Fanandrana. Statistical analysis revealed a significant difference in prevalence of *A. cantonensis* worms in *Achatina* snails collected in those localities (χ^2^ = 38.343, degrees of freedom = 2; p<0.0001). 

Our data report an overall prevalence of 26.9% of *A. cantonensis* worms in *Achatina* snails from the east coast of Madagascar, which is higher than in other *A. cantonensis*–endemic regions such as China (21.5%) (*8*), Brazil (21.7%) (*9*), and Ecuador (15.2%) (*10*). The high prevalence of *A. cantonensis* worms in the *Achatina* genus demonstrates their involvement in the life cycle of this zoonotic parasite in Madagascar. Furthermore, 2 *R. norvegicus* rats (a juvenile male and pregnant female) caught in Ankirihiry were parasitized by *A. cantonensis* worms*.* The presence of this lungworm in *R. norvegicus* rats and snails in Ankirihiry suggest an active circulation of this parasite and a potential risk for human neuroangiostrongyliasis on the east coast of Madagascar because of the abundance of hosts (intermediate or definitive) and the consumption of either infected snails or food contaminated by infective larva. Properly boiling water to cook snail meat thoroughly before consumption is critical to avoid infection. Because *Rattus* spp. rats are found everywhere in Madagascar, future research should investigate other locations to survey the prevalence of *A. cantonensis* worms in rats, as well as the distribution of *Achatina* spp. snails and the prevalence of the parasite in those and other snail species to implement surveillance. Although no human infections have been reported in Madagascar, our findings indicate active circulation of the *A. cantonensis* rat lungworm, posing a potential zoonotic risk.

Appendix Additional information about *Angiostrongylus cantonensis* lungworms in definitive and intermediate hosts, Madagascar, 2024
