# Supplementary material for: Angiostrongylus cantonensis Lungworms in Definitive and Intermediate Hosts, Madagascar, 2024
Source: Emerg Infect Dis. 2025 Oct;31(10):2054–6. doi: 10.3201/eid3110.241741 (PMC12483016; doi:10.3201/eid3110.241741)
Supplement: Appendix — Additional information about Angiostrongylus cantonensis lungworms in definitive and intermediate hosts, Madagascar, 2024 [file 24-1741-Techapp-s1.pdf]

Article DOI: https://doi.org/10.3201/eid3110.241741

EID cannot ensure accessibility for supplementary materials supplied by authors. Readers who have difficulty accessing supplementary content should contact the authors for assistance.

Angiostrongylus cantonensis Lungworms in Definitive and Intermediate Hosts, Madagascar, 2024

Appendix

Supplementary data

Rat and Snail sampling

Rats were captured in March 2024 at three communes: Fanandrana (18.2858°S, 49.1218°E, rural commune), Antetezambaro (18.0137°S, 49.4021°E, rural commune) and Ankirihiry (18.1392°S, 49.3995°E, urban commune) in Toamasina district. Rats were captured using wire mesh live traps (Besançon Technical Service, 30L × 10W × 10H cm, BTS Company, Besançon, France) and Sherman traps (23L × 7.5W × 9H cm, H.B. Sherman Traps Inc., Tallahassee, Florida, USA). Each trap was baited with peanut butter with the bait replaced every day. Rats were trapped on three successive nights at each study site. All captured rats were euthanized by cervical dislocation. Ectoparasites were removed by brushing the fur of each individual within a 30 cm deep basin and preserved in ethanol. Body measurements, weight and reproductive status characteristics of rats were taken to facilitate identification of species and age estimation. Immediately after death, blood samples were taken by cardiac puncture and preserved in blotting paper within an Eppendorf tube. The whole lung of captured individuals was removed and dissected under binocular magnifier to isolate lungworms and preserved in 95% ethanol before molecular analysis.

Sampling of snails was carried out at the three study sites (Fanandrana, Antetezambaro, Ankirihiry) in Toamasina districts. The foot tissue of each snail was preserved in 95% ethanol for molecular analysis of *Angiostrongylus* larvae.

Molecular identification of rat lungworm

DNA extraction from rat lungworm and snail foot tissue was carried out according to the manufacturer's protocol using the commercial DNeasy Blood and Tissue kit (Qiagen, Germany). Eluted DNA was stored at −20°C before amplification. Conventional PCR targeting cytochrome C gene was performed on DNA eluted from two individual lungworms, using the following set of primers: 5′-TTAGTTTRCATTGTGCTGG-3′ and 5′-CATCAAAGACTAATACCAG- 3′ (*1*) to confirm their molecular identity and assess the genetic diversity of *Angiostrongylus cantonensis*. The amplicons obtained were migrated on a 1.5% agarose gel under a 120-V generator for 90 minutes. PCR products were visualized by Gelscan Vilber and subsequently sent for Sanger sequencing to Genoscreen (Lille, France) for bioinformatics analysis.

Phylogenetic analysis and accession numbers

The two nucleotide sequences obtained were manually edited using *Geneious Prime* sequence alignment editor version 2024.1 (*2*). The program Basic Local Alignment Search Tool (BLAST, https://blast.ncbi.nlm.nih.gov) (*3*) was used to compare identified nucleotide sequences with those available in the NCBI Genbank. Before performing the phylogenetic analysis, the best substitution model was calculated in *jModelTest* 2.1.10 using the lowest Akaike information criterion score (*4*). The phylogenetic trees were constructed using the Tamura-Nei model with the maximum likelihood method. Bootstrap analyses were performed with 10,000 iterations using *MEGA X* software (*5*).

Appendix Table 1. Accession numbers of *A. cantonensis* sequences used

Country	*A. cantonensis* host	Accession number
Madagascar	*Rattus norvegicus*	PV185895
Madagascar	*Rattus norvegicus*	PV185896
Spain	*Rattus rattus*	PP748576
Spain	*Rattus norvegicus*	PP748575
Spain	*Rattus norvegicus*	PP748573
Spain	*Rattus norvegicus*	PP748572
Spain	*Rattus rattus*	PP748571
Australia	*Rattus rattus*	MK570629
USA	*Rattus rattus*	MH069734
Brazil	*Achatina fulica*	JX471056
Brazil	*Rattus norvegicus*	GU138111
Japan	*Rattus rattus*	AP017672
Thailand	*Rattus norvegicus*	KU532144
Guadeloupe	*Rattus rattus*	OQ255912
Guadeloupe	*Rattus rattus*	OQ255913
Guadeloupe	*Rattus rattus*	OQ255914
Guadeloupe	*Rattus rattus*	OQ255915
Guadeloupe	*Rattus rattus*	OQ255916
Guadeloupe	*Rattus rattus*	OQ255917
Guadeloupe	*Rattus rattus*	OQ255918
Guadeloupe	*Rattus norvegicus*	OQ255919
People's Republic of China	*Rattus norvegicus*	AB684364
People's Republic of China	*Rattus norvegicus*	AB684365
Myanmar	*Achatina fulica*	KU532145

qPCR screening of *Angiostrongylus cantonensis*

Molecular screening of *Angiostrongylus cantonensis* was performed by TaqMan qPCR targeting Internal Transcribed Spacer (ITS) gene according to Qvarnstrom's protocol (*6*), using the following set of primers and probe: AcanITS1F1 (5′-TTCATGGATGGCGAACTGATAG-3′), AcanITS1R1 (5′-GCGCCCATTGAAACATTA TACTT-3′) and TaqMan probe AcanITS1P1 (5′-6-carboxyfluorescein-ATCGCATATCTACTATACGCATGTGACACCTG-BHQ-3′). DNA from a rat lungworm confirmed as *A. cantonensis* was used as a positive control and nuclease free water was used as the negative control.

Appendix Table 2. Detection of *Angiostrongylus cantonensis* in rats (N: Number of individuals tested)

Locality	Fanandrana		Antetezambaro		Ankirihiry			
Definitive	*R. rattus*		*R. rattus*		*R. rattus*		*R. norvegicus*	
host	N	Infected	N	Infected	N	Infected	N	Infected
Sex								
Female	9	0	15	0	5	0	1	1/1
Male	12	0	24	0	11	0	1	1/1
Age								
Juvenile	9	0	22	0	11	0	1	1/1
Adult	12	0	17	0	5	0	1	1/1
Total	21	0/21	39	0/39	16	0/16	2	100

Appendix Table 3. Detection of *Angiostrongylus cantonensis* in snails (N: Number of individuals tested)

Locality	Fanandrana		Antetezambaro		Ankirihiry	
Intermediate host	*Achatina* spp.		*Achatina* spp.		*Achatina* spp.	
	N	Infected	N	Infected	N	Infected
Total	43	2/43 (4.6)	54	16/54 (29.6)	33	17/33 (51.5)

Ethical statements

The rodent sampling carried out was completed through surveillance in collaboration with the Ministry of Public health in Madagascar. This collaborative agreement aims to reduce the density of plague reservoirs in Madagascar; we did not need specific authorization for the sampling. During this work, individual rats were manipulated and euthanized in accordance with guidelines accepted by the scientific community for the handling of wild animals (*7*), the directive 2010/63/EU of the European Parliament and approved by the local ethics committee of the Institut Pasteur de Madagascar.

The senior author on this project Beza Ramasindrazana is a small mammal's specialist in Madagascar and is a member of the animal ethics at Institut Pasteur de Madagascar. He also has the certificate in animal experimentation (since 2024 after a specific training).

For the snails, local populations are using them for food as the species is found everywhere in the region of Toamasina.

References

1. Gamiette G, Ferdinand S, Couvin D, Dard C, Talarmin A. The recent introduction of *Angiostrongylus cantonensis* and its intermediate host *Achatina fulica* into Guadeloupe detected by phylogenetic analyses. Parasit Vectors. 2023;16:276. PubMed https://doi.org/10.1186/s13071-023-05872-4

2. Geneious Prime. 2024.1.1, Biomatters Ltd., Auckland, New Zealand [cited 2024 Jul 12]. https://www.geneious.com

3. Altschul SF, Gish W, Miller W, Myers EW, Lipman DJ. Basic local alignment search tool. J Mol Biol. 1990;215:403–10. PubMed https://doi.org/10.1016/S0022-2836(05)80360-2

4. Darriba D, Taboada GL, Doallo R, Posada D. jModelTest 2: more models, new heuristics and parallel computing. Nat Methods. 2012;9:772. PubMed https://doi.org/10.1038/nmeth.2109

5. Kumar S, Stecher G, Li M, Knyaz C, Tamura K. MEGA X: Molecular Evolutionary Genetics Analysis across computing platforms. Mol Biol Evol. 2018;35:1547–9. PubMed https://doi.org/10.1093/molbev/msy096

6. Qvarnstrom Y, da Silva ACA, Teem JL, Hollingsworth R, Bishop H, Graeff-Teixeira C, et al. Improved molecular detection of *Angiostrongylus cantonensis* in mollusks and other environmental samples with a species-specific internal transcribed spacer 1-based TaqMan assay. Appl Environ Microbiol. 2010;76:5287–9. PubMed https://doi.org/10.1128/AEM.00546-10

7. Sikes RS; Animal Care and Use Committee of the American Society of Mammalogists. 2016 Guidelines of the American Society of Mammalogists for the use of wild mammals in research and education. J Mammal. 2016;97:663–88. PubMed https://doi.org/10.1093/jmammal/gyw078
